# Pituitary adenoma secondary to primary hypothyroidism: Two case reports

**DOI:** 10.1097/MD.0000000000019222

**Published:** 2020-02-21

**Authors:** Jianyang Du, Hang Ji, Jiaqi Jin, Shuai Gao, Xiuwei Yan, Shaoshan Hu

**Affiliations:** Department of Neurosurgery, The Second Affiliated Hospital of Harbin Medical University, Harbin 150086, China.

**Keywords:** case report, pituitary adenoma, primary hypothyroidism, surgical resection

## Abstract

**Rationale:**

Primary hypothyroidism is characterized by loss of thyroxine feedback inhibition and overproduction of thyrotropin-releasing hormone, which might result in reactive pituitary hyperplasia. However, pituitary adenoma secondary to primary hypothyroidism is extremely rare and usually underdiagnosed, and the pathogenic mechanism remains unclear. Herein, we reported two cases with pituitary adenoma secondary to primary hypothyroidism.

**Patient concerns:**

Case 1: A 35-year-old man presented to the local clinic with a 2-year history of fatigue, puffiness in the bilateral lower extremities and facial region, and coarseness of facial features. Additionally, his relatives also supplemented that he suffered from hypomnesis and hypophrenia.

Case 2: A 56-year-old, postmenopausal woman presented to the local clinic with fatigue, dry skin, and sluggishness.

**Diagnoses:**

The pathological diagnosis of two patients was plurihormonal pituitary adenoma.

**Interventions:**

A microscopical tumorectomy was performed when the two patients were admitted to our hospital. Thyroid hormone replacement therapy (thyroxine 50 μg/day) was prescribed after microsurgery.

**Outcomes:**

After 32 months (Case 1) or 43 months (Case 2) follow-up respectively, there was no recurrence, and the symptoms were completely relieved.

**Lessons:**

Pituitary hyperplasia caused by primary hypothyroidism responds well to thyroid hormone replacement therapy. It is worth noting that repeated detection of serum T3, T4, and thyroid-stimulating hormone (TSH) should be performed 3 months after replacement therapy. If the results showed that TSH level decreased partly, while thyroid function did not improve significantly, long-term increased secretion of pituitary TSH adenoma should be considered. And microsurgical resection via a transsphenoidal approach could be ordered. If the optic nerve or optic chiasm were pressed by the adenoma, microsurgery should be performed to relieve the pressure immediately. And then, thyroxine tablet substitute therapy should be performed after surgery.

## 1 Introduction

Primary hypothyroidism is characterized by loss of thyroxine feedback inhibition and overproduction of thyrotropin releasing hormone (TRH), which might result in reactive pituitary hyperplasia.^[1–5]^ However, pituitary adenoma secondary to primary hypothyroidism is extremely rare and usually underdiagnosed.^[6,7]^ The pathogenic mechanism of this entity remains unclear, and the clinical characteristics, as well as the therapeutic regimen, have not yet to be well elucidated.

In the current study, we reported two cases with pituitary adenoma secondary to primary hypothyroidism, and relevant literature was reviewed.

## 2 Case report

This study was approved by the Ethics Committee and Institutional Review Board of the Second Affiliated Hospital of Harbin Medical University. The two patients have provided informed consent for publication of the case.

### 2.1 Case 1

**Figure 1:**
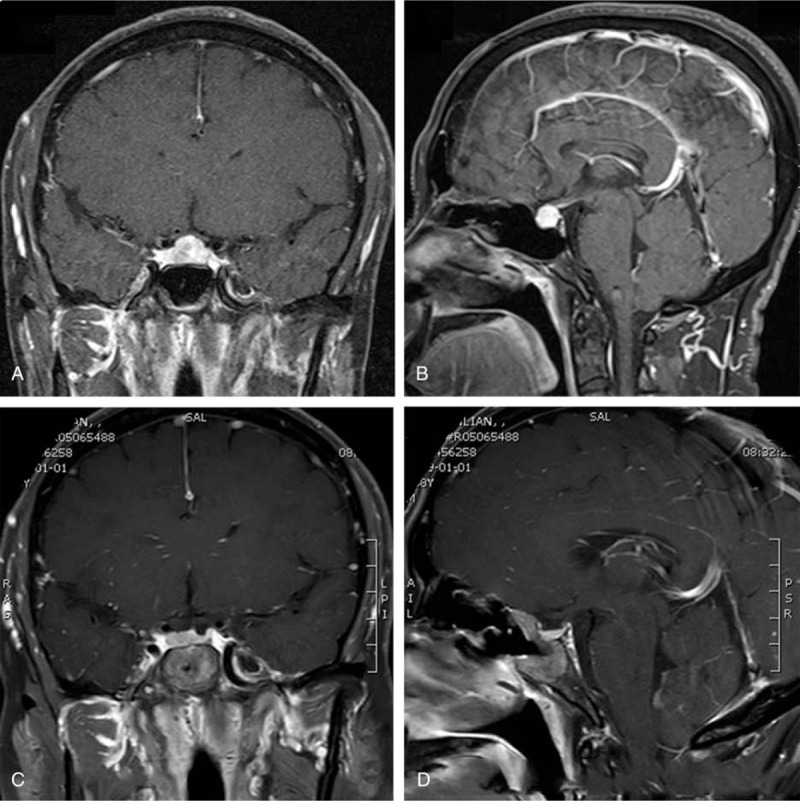
Radiological profiles of Case 1. Intracranial MRI of the patient after treatment (A) coronal and (B) sagittal contrast-enhanced T1-weighted MRI revealed a 1.8 × 1.4 × 1.3 cm sellar mass with remarkable enhancement, and the optic chiasma was oppressed. (C and D) Postoperative MRI showed the mass was partially resected. The optic chiasma is in good shape. MRI = magnetic resonance imaging.

A 35-year-old man presented to the local clinic with a 2-year history of fatigue, puffiness in the bilateral lower extremities and facial region, and coarseness of facial features. Additionally, his relatives also supplemented that he suffered from hypomnesis and hypophrenia. Color Doppler ultrasound showed a diffuse goiter. Oral thyroxine (100 μg/day) was prescribed but exhibited no significant benefit. Ten days prior to admission to our department, he developed bilateral conjunctival congestion. Magnetic resonance imaging (MRI) revealed a 1.8 × 1.4 × 1.3 cm irregular mass in the sellar region, and the optic chiasma was compressed. After the administration of Gd-diethylenetriamine pentaacetic acid (Gd-DTPA), the mass was remarkably enhanced (Fig. 1). Endocrinological examination showed elevated thyroid-stimulating hormone (TSH = 100 μIU/mL; normal 0.27–4.20 μIU/mL), reduced free T3 (FT3 = 1.24 pmol/L; normal 3.1–6.8 pmol/L), and reduced free T4 (FT4 = 2.04 pmol/L; normal 12–22 pmol/L); the serum prolactin (PRL) level was slightly elevated (800.12 μIU/mL; normal 55–278 μIU/mL). A diagnosis of pituitary adenoma secondary to primary hypothyroidism was suspected. A microscopical tumorectomy was performed. Postoperatively, the hormone levels were gradually improved (Table 1). Histopathological examination showed a plurihormonal pituitary adenoma. Thyroid hormone replacement therapy (thyroxine 50 μg/day) was prescribed after microsurgery. During the follow-up period of 32 months, there was no recurrence, and the symptoms were completely relieved. Now, patients orally take thyroxine 25 μg/day, and there are no signs of recurrence of related symptoms.

**Table 1:**
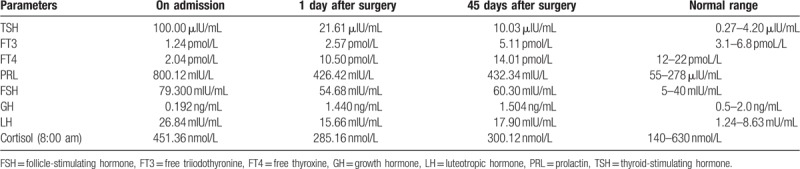
The hormone levels in Case 1.

### 2.2 Case 2

**Figure 2:**
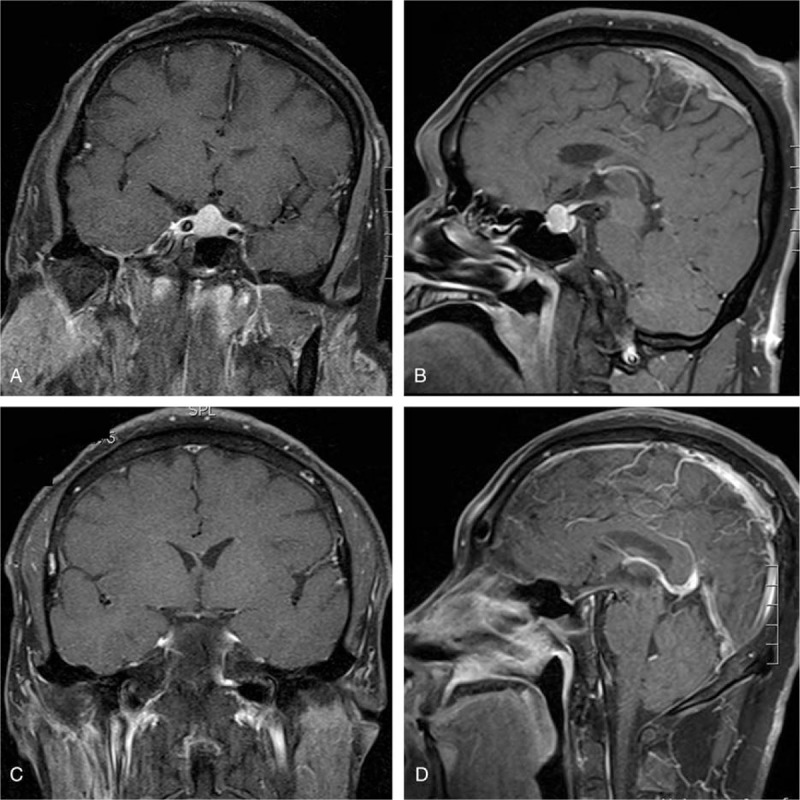
Radiological profiles of Case 2. Intracranial MRI of the patient before treatment (A) coronal and (B) sagittal showing a pituitary mass obviously and uniformly enhanced by Gd-EDTA injection. The upper edge of the mass is round, and optic nerve or optic chiasm were pressed by the mass. (C and D) MRI coronal and sagittal scan showing pituitary return to normal size after taking surgery. MRI = magnetic resonance imaging.

**Figure 3:**
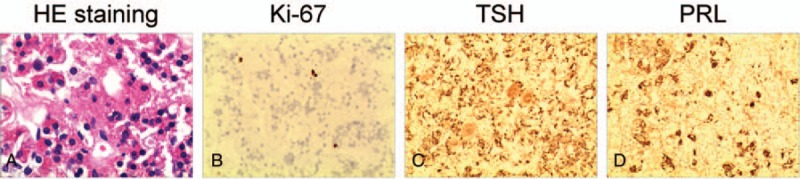
Pathological diagnosis of Case 2. Acinar-like cell masses of different sizes are diffusely distributed, and some acinar cells are enlarged and proliferated, and the nucleus is (A) round or elliptical. Immunohistochemistry showed partial expression of (B) Ki-67 and high expression of (D) PRL and (C) TSH. The final pathological diagnosis is plurihormonal pituitary adenoma. PRL = prolactin, TSH = thyroid-stimulating hormone.

A 56-year-old, postmenopausal woman presented to the local clinic with fatigue, dry skin, and sluggishness. In the local clinic, she was diagnosed with hypothyroidism. And oral thyroxine (100 μg/day) was administered for 3 years but exhibited no significant benefit. In recent days, she developed a headache and visual disturbance. MRI showed a sellar mass with remarkable enhancement, and the mass compressed the optic chiasma (Fig. 2). Endocrinological examination showed elevated TSH (100 μIU/mL; normal 0.27–4.20 μIU/mL), reduced FT3 (2.35 pmol/L; normal 3.1–6.8 pmol/L), and reduced FT4 (5.62 pmol/L; normal 12–22 pmol/L); the serum PRL level was slightly elevated (723.12 μIU/mL; normal 55–416 μIU/mL). A diagnosis of pituitary adenoma secondary to primary hypothyroidism was suspected. A tumorectomy was performed via a transnasal-sphenoidal approach. Postoperatively, the hormone levels were gradually improved (Table 2). Histopathological examination showed a plurihormonal pituitary adenoma. It showed that acinar-like cell masses of different sizes were diffusely distributed, and some acinar cells were enlarged and proliferated. And the nucleus was round or elliptical (Fig. 3A). Immunohistochemistry showed partial expression of Ki-67 and high expression of PRL and TSH (Fig. 3B–D). Thyroid hormone replacement therapy (thyroxine 50 μg/day) was administered after microsurgery. After a 43-month follow-up, no recurrence was noted, and the symptoms were completely relieved. There was no recurrence of the symptoms associated with the sign.

**Table 2:**
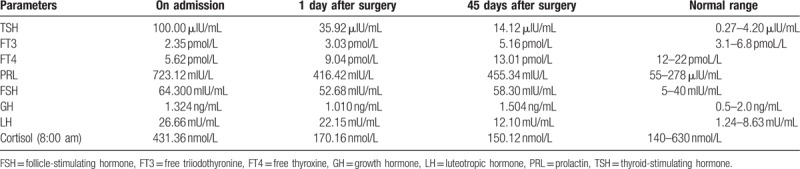
The hormone levels in Case 2.

## 3 Discussion

In 1985, Scheithauer et al performed histopathological and immunocytological examinations of pituitary glands in the autopsy of 64 patients with long-standing primary hypothyroidism, and they noted pituitary enlargement in 91% cases, pituitary tumorous hyperplasia in 12% cases, and pituitary adenoma in 18.7% cases.^[8]^ The authors also proposed that pituitary tumorous hyperplasia might represent an intermediate stage between enlargement and adenoma. Pituitary hyperplasia refers to non-neoplastic growth or enlargement of pituitary gland cells without qualitative changes in cell biology, which include physiological, pathological, syndrome-related (such as Addison disease, Klinefelter syndrome, and Turner syndrome) and idiopathic subtypes.^[9]^ Pituitary hyperplasia caused by primary hypothyroidism is due to the loss of thyroxine feedback inhibition to the hypothalamus, which can induce the overproduction of TRH and hyperplasia of lactotrophs. Moreover, patients with pituitary hyperplasia or pituitary adenoma may as well be asymptomatic. Noteworthily, pituitary hyperplasia has no histopathological atypia or pathologic mitosis, and hormone replacement therapy can be effective.^[10–13]^ However, pituitary adenoma is a benign neoplasm originating from anterior pituitary cells with necrosis and/or cystic changes.^[7,14]^ We speculate that pituitary adenoma shares similar pathogenetic mechanisms with pituitary tumorous hyperplasia: long-term stimulation by TRH and loss of thyroxine feedback inhibition. Pituitary gland cells are adenomatous hyperplasia. Pituitary adenomas exhibit a typical structural disorder, cell necrosis, and cystic changes. Immunohistochemistry showed that more than two hormones were positive. Most patients require surgery, postoperative combined hormone therapy.

In the current study, we also noted hyperprolactinemia in these two cases. According to literature, pituitary adenomas secondary to primary hypothyroidism are usually associated with elevated PRL levels. In 1989, Ahmed et al reported five cases of pituitary enlargement secondary to primary hypothyroidism, in which endocrinological examination showed both elevated TSH and PRL; the hormone levels returned to normal following thyroid hormone replacement therapy, indicating the hyperplasia of TSH- and PRL-secreting cells is reversible, and thus the authors considered this condition as ‘pseudo-prolactinoma syndrome’.^[13]^ Previous studies showed the occurrence rate of hyperprolactinemia in Hashimoto thyroiditis and primary hypothyroidism is 11.1% and 21% to ∼42.4%, respectively.^[15–17]^ Thus, in female patients, hypothyroidism can manifest as lactation, amenorrhea, and infertility.^[18]^ The PRL levels in patients with hypothyroidism usually range from 1680 mIU/mL to 2120 mIU/mL, which are lower than those in patients with prolactinoma. For patients presenting with lactation or amenorrhea, the thyroid hormone examinations should be highlighted for identifying the underlying hypothyroidism. The pathogenesis of hyperprolactinemia in hypothyroidism remains unclear; the mainstream hypothesis is that TRH can not only activate the TSH-secreting cells but also stimulate the PRL-secreting cells, and the enlarged pituitary gland compresses the pituitary stalk affecting the hypophysioportal circulation and resulting in reduced dopamine (an antagonist against the PRL releasing hormone).

Several groups of cases have shown that pituitary hyperplasia has characteristic features on computed tomography. When the pituitary hyperplasia, the upper edge can be pointed, and the upper edge of the pituitary adenoma is round. MRI showed similar results.^[19]^ In fact, our patient's MRI showed a lump with a pointed round edge.

Pituitary hyperplasia caused by primary hypothyroidism responds well to thyroid hormone replacement therapy.^[10–13,20]^ It is worth noting that repeated detection of serum T3, T4, and TSH should be performed 3 months after replacement therapy. If the results showed that TSH level decreased partly, but thyroid function did not improve significantly, long-term increased secretion of pituitary TSH adenoma should be considered, microsurgical resection via a transsphenoidal approach could be ordered. If the optic nerve or optic chiasm were pressed by the adenoma, microsurgery should be chosen first, to relieve the pressure, and thyroxine tablet substitute therapy should be taken after surgery.^[21]^ In the current study, both patients showed no endocrinological improvement following administration of long-term oral thyroxin while the thyroid hormone levels were improved after surgery combined with oral thyroxin. Especially, for the cases with optic chiasma compression, surgical resection of the adenoma is necessary for visual restoration. Thus, the differential diagnosis between pituitary adenoma and pituitary hyperplasia is crucial, and microsurgical resection via a transsphenoidal approach should be reserved for decompression of the optic chiasm or pathological diagnosis, in the case of a pituitary mass not responding to, or worsening on, thyroid hormone replacement therapy.

Pituitary adenoma secondary to primary hypothyroidism is an extremely rare disorder, and the neurosurgeons should be aware of this entity as it is usually underdiagnosed. Surgical resection combined with thyroid hormone replacement therapy should be highlighted, which can lead to a favorable prognosis.

## Author contributions

**Conceptualization:** Jianyang Du.

**Data curation:** Jianyang Du, Shuai Gao.

**Formal analysis:** Jianyang Du, Hang Ji.

**Funding acquisition:** Jianyang Du, Xiuwei Yan, Shaoshan Hu.

**Investigation:** Jianyang Du, Hang Ji.

**Methodology:** Jianyang Du, Xiuwei Yan.

**Project administration:** Jianyang Du, Jiaqi Jin.

**Resources:** Jianyang Du, Jiaqi Jin, Xiuwei Yan.

**Software:** Jianyang Du.

**Supervision:** Jianyang Du, Hang Ji, Jiaqi Jin.

**Validation:** Jianyang Du.

**Visualization:** Jianyang Du, Shaoshan Hu.

**Writing – original draft:** Jianyang Du, Shaoshan Hu.

**Writing – review & editing:** Jianyang Du, Shaoshan Hu.

Jianyang Du: 0000-0001-9682-4183.

Jianyang Du orcid: 0000-0001-9682-4183.
